# Ecotoxicological Impacts of Perfluorooctane Sulfonate on the Freshwater Snail *Lanistes carinatus*: Oxidative Stress, Neurotoxicity, and Histopathological Alterations

**DOI:** 10.3390/ijms26188898

**Published:** 2025-09-12

**Authors:** Mohamed Hamed, Mohammed Abdel-Wahab, Rashad E. M. Said, Alaa El-Din H. Sayed

**Affiliations:** 1 Department of Comparative Biomedical Sciences, School of Veterinary Medicine, Louisiana State University, Skip Bertman Drive, Baton Rouge, LA 70803, USA; 2 Department of Zoology, Faculty of Science, Al-Azhar University (Assiut Branch), Assiut 71524, Egypt; mnassar919@azhar.edu.eg (M.A.-W.); banjawy@yahoo.com (R.E.M.S.); 3 Department of Zoology, Faculty of Science, Assiut University, Assiut 71516, Egypt; alaasayed@aun.edu.eg; 4 Molecular Biology Research & Studies Institute, Assiut University, Assiut 71516, Egypt

**Keywords:** PFOS, *Lanistes carinatus*, ecotoxicity, antioxidant activity, neurotoxicity, histopathological alterations

## Abstract

Perfluorooctane sulfonate (PFOS), which is known for its environmental persistence and bioaccumulation, poses substantial impacts to aquatic ecosystems. This study assesses the toxic effects of PFOS in the freshwater snail *Lanistes carinatus* using biomarkers for antioxidant activity, neurotoxicity, and tissue damage. Snails exposed to PFOS (1, 3, 10 mg/L for 14 days) displayed lipid peroxidation (LPO) levels that increased by 16.3–67.5%, and malondialdehyde (MDA) levels that rose by 10.0–58.4%, indicating oxidative damage. Enzyme activities for glutathione S-transferase (GST), glutathione peroxidase (GPx), and catalase (CAT) increased, ranging from 10.0 to 58.3%, 10.0 to 58.4%, and 10.0 to 58.4%, respectively, whereas levels of reduced glutathione (GSH) dropped by 15.0–41.5% and Superoxide dismutase (SOD) decreased by 15.0–41.4%. The activity of acetylcholinesterase (AchE) was reduced by a range of 15.0–40.0%, suggesting neurotoxic effects. Histopathological changes in the digestive gland were also noted. Further research on the effects of PFOS on mollusks is required, and investigation into sex-specific toxicity is needed. This shed light on *L. carinatus* as a sentinel species, providing helpful information for the monitoring and regulation of PFOS in aquatic environments.

## 1. Introduction

Per- and polyfluoroalkyl substances (PFASs) are synthetic molecules used in consumer and industrial environments around the world. The variety of PFAS found in the environment is on the increase. About 9000 chemical substances listed by the USEPA in 2021 increased to over 12,000 by 2023 [1]. The persistence of PFAS in the environment and their ability to accumulate in living organisms make them widespread global pollutants linked to ecological and human health hazards [2,3,4].

A vast range of products and human needs including semiconductors, surfactants, cosmetics, food packing, etc., extensively use PFAS [5,6,7]. The widespread presence of these compounds is due to the unique carbon–fluorine (C-F) bonds they contain, which impart exceptional resistance to oil, heat, and water [8,9]. The same properties drive the persistence and bioaccumulation potential of PFAS in different biotopes and organisms including humans [10,11,12]. As a result, its widespread use and environmental stability have led to chronic adverse impacts on ecosystems and public health [9,13,14,15].

Regulatory attention continues to be focused primarily on specific PFAS, such as perfluorooctanoic acid (PFOA) and perfluorooctane sulfonate (PFOS), even though there are thousands of documented compounds. Globally, PFOA/PFOS are extensively studied because they persist, can travel over long distances, are found in different environmental samples, living organisms, and humans, and have been shown to disrupt immune, metabolic, and endocrine systems [16,17,18,19]. Notably, PFOS contaminates surface waters [20,21,22], wastewater [23,24], and humans at a concentration of mg/L in affected communities [25,26]. The regulatory–environmental disconnect has driven an increase in ecological research [27,28,29,30].

Human exposure to PFOS was associated with hepatic damage, thyroid problems, reproductive inhibition, developmental toxicity, and elevated cholesterol [31,32,33,34], and long-term exposure has been linked to a potential increased risk of cancer [35]. PFOS causes harm to wildlife’s organs, reproductive systems, and metabolism [36,37]. Invertebrates, which are vital to ecosystem health because they play a crucial role in cycling nutrients and food chains, are experiencing population declines caused by pollution resulting from human activities [38,39]. The vulnerability of PFOS highlights the need to study its ecotoxicity, as invertebrate biomass loss directly threatens the multifunctionality of ecosystems [40].

PFOS tends to accumulate in aquatic organisms through respiration and consumption, often present at low concentrations (0.2–100 ng/L) across various environmental media [41]. Oxidative stress is driven by this amplification, which disrupts antioxidant defenses and detoxification pathways [42,43], primarily through impaired nuclear receptor signaling, fatty acid β-oxidation, and mitochondrial permeability [44]. Mechanistically, PFOS disrupts the normal functioning of detoxification, lipid metabolism, and xenobiotic processing pathways [45,46]. In *Daphnia carinata*, PFOS causes acute effects, with a 48 h LC_50_ of 8.8 mg/L, which is more toxic than PFOA (78.2 mg/L) [47].

Snails and other sentinel organisms can accumulate persistent pollutants and metals, making them useful for monitoring the effects of contaminants on development [48,49]. Their heightened sensitivity to pollutants helps in early detection of environmental stress [50]. *L*. *carinatus*, a widely distributed freshwater snail in Egypt, mostly inhabiting still water environments, serves as an example for monitoring aquatic pollution [51,52]. The selection of this animal was based on local abundance, ease of collection, and laboratory hardiness. It is essential to comprehend invertebrate toxicity, as its negative effects have a ripple effect throughout food chains. Through examining their responses to contaminants, more comprehensive ecological results can be predicted. Oxidative stress defenses in cells rely on the coordinated action of crucial biomarkers, such as lipid peroxidation (LPO) and malondialdehyde (MDA), as well as enzymes like glutathione transferase (GST), catalase (CAT), glutathione peroxidase (GPx), reduced glutathione (GSH), and superoxide dismutase (SOD).

These biomarkers play a crucial role in reducing oxidative impact and are well-employed indicators of contamination-induced stress [53,54]. Exposure to PFOS has a substantial impact on antioxidant activity [55], which can be monitored through fluctuations in SOD, CAT, and GPx levels. Prolonged exposure to PFOS in earthworms leads to increased levels of malondialdehyde (MDA), a byproduct of lipid peroxidation that indicates the accumulation of ROS [56]. A typical ROS surge in this context results in a biphasic antioxidant response, characterized by elevated records of SOD, POD, CAT, and GPx, which then decrease. In line with this pattern, PFAS causes oxidative stress across aquatic species [57,58], as seen in increased SOD/CAT/GR activities in *Oreochromis niloticus* hepatocytes [59] and higher MDA in *Pimephales promelas* [60].

A large body of research has confirmed the toxicity of PFAS in aquatic organisms, particularly fish, resulting in cellular and molecular disruptions [58,61]. Invertebrate responses to pollutants have a major role in determining the viability of aquatic ecosystems. Despite this, there is a lack of PFOS toxicity data for key invertebrates, particularly snails such as *L. carinatus*, making an urgent evaluation necessary. Thus, the main goal of the current research was to assess PFOS (1, 3, and 10 mg/L) toxicity in the freshwater snail *L. carinatus,* following 14 days of exposure. Multiple oxidative stress markers (LPO, MDA, GST, CAT, GPx, GSH, and SOD), acetylcholinesterase activity as a neurotoxicity indicator, along with histopathological examinations in the digestive gland, were all assessed.

## 2. Results

### 2.1. Antioxidant Defense Enzymes

**Figure 1:**
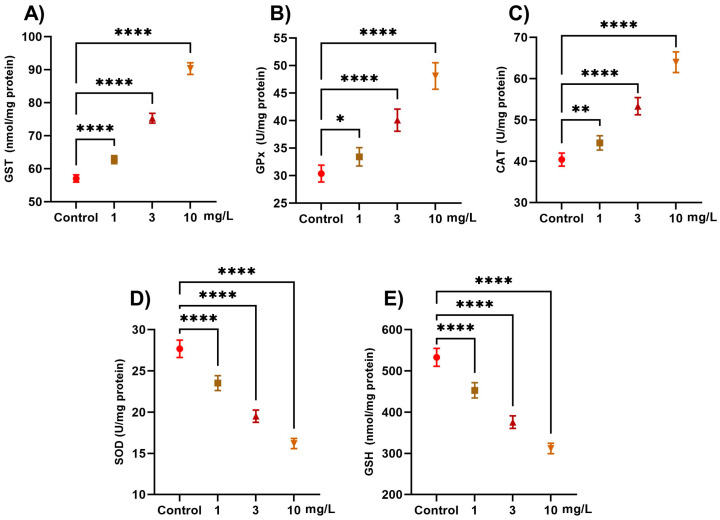
Effect of PFOS exposure on antioxidant enzymes and glutathione levels in *L. carinatus*. Snails were exposed to PFOS at concentrations of 1, 3, and 10 mg/L. (**A**) Glutathione S-transferase (GST, nmol/mg protein), (**B**) glutathione peroxidase (GPX, U/mg protein), (**C**) catalase (CAT, U/mg protein), (**D**) superoxide dismutase (SOD, U/mg protein), and (**E**) reduced glutathione (GSH, nmol/mg protein). Data are presented as the mean ± SD of six biological replicates. Statistical significance compared with the control: *p* < 0.05 (*), *p* < 0.01 (**), and *p* < 0.0001 (****).

Exposure to PFOS caused significant alterations in antioxidant defense enzymes in snails (Figure 1) compared with non-exposed groups. GST activity increased by 10.0% at 1 mg/L, 31.9% at 3 mg/L, and 58.3% at 10 mg/L, compared with the control group (*p* < 0.0001). GPx levels increased by 10.0% at 1 mg/L, 31.9% at 3 mg/L, and 58.4% at 10 mg/L, when compared with the control (*p* < 0.0001). CAT activity also increased significantly, with a 10.0% increase at 1 mg/L, 32.0% at 3 mg/L, and 58.4% at 10 mg/L, relative to the control (*p* < 0.0001). GSH levels declined significantly compared with the control (*p* < 0.0001), showing a 15.0% decrease at 1 mg/L, 29.2% at 3 mg/L, and 41.5% at 10 mg/L, reflecting antioxidant depletion. SOD activity also decreased, with reductions of 15.0% at 1 mg/L, 29.4% at 3 mg/L, and 41.4% at 10 mg/L, in comparison with the control (*p* < 0.0001).

### 2.2. Oxidative Stress Markers

**Figure 2:**
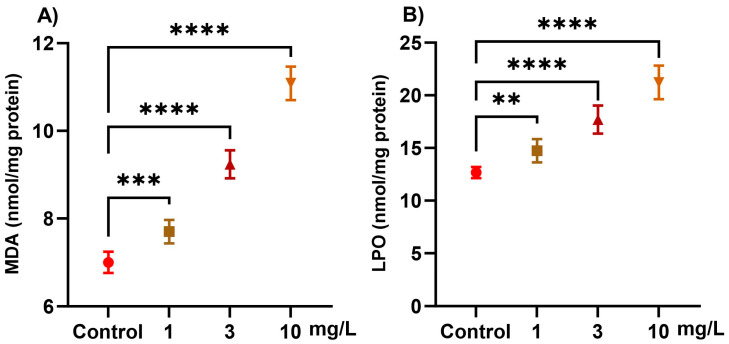
Effect of PFOS exposure on oxidative stress markers in *L. carinatus* snails were exposed to PFOS at concentrations of 1, 3, and 10 mg/L. (**A**) Malondialdehyde (MDA, nmol/mg protein) and (**B**) lipid peroxides (LPO, nmol/mg protein). Data are presented as mean ± SD of six biological replicates. Statistical significance compared with the control: *p* < 0.01 (**), *p* < 0.001 (***), and *p* < 0.0001 (****).

Exposure to PFOS induced significant, dose-dependent (1, 3, and 10 mg/L) alterations in oxidative stress markers in snails (Figure 2). LOP increased progressively with PFOS exposure, showing a 16.3% increase at 1 mg/L, 39.6% at 3 mg/L, and 67.5% at 10 mg/L, compared with the control (*p* < 0.0001). MDA, a marker of lipid peroxidation, was elevated by 10.0% at 1 mg/L, 32.0% at 3 mg/L, and 58.4% at 10 mg/L, relative to the control group (*p* < 0.0001).

### 2.3. Neurotoxicity Markers

**Figure 3:**
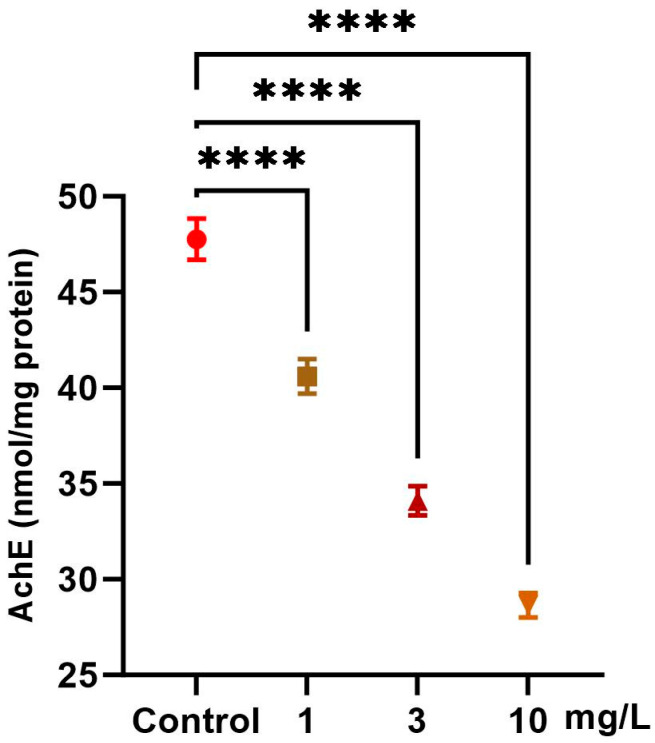
Effect of PFOS exposure on acetylcholinesterase activity (AchE) in *L. carinatus*. Snails were exposed to PFOS at concentrations of 1, 3, and 10 mg/L. Data are presented as mean ± SD of six biological replicates. Statistical significance compared with the control: *p* < 0.0001 (****).

Exposure to increasing concentrations of PFOS (1, 3, and 10 mg/L) induced significant, dose-dependent alterations in the neurotoxicity marker in snails (Figure 3). AchE activity significantly decreased relative to the control by 15.0% at 1 mg/L, 28.6% at 3 mg/L, and 40.0% at 10 mg/L (*p* < 0.0001), indicating potential neurotoxic effects.

### 2.4. Histology of the Digestive Gland

**Figure 4:**
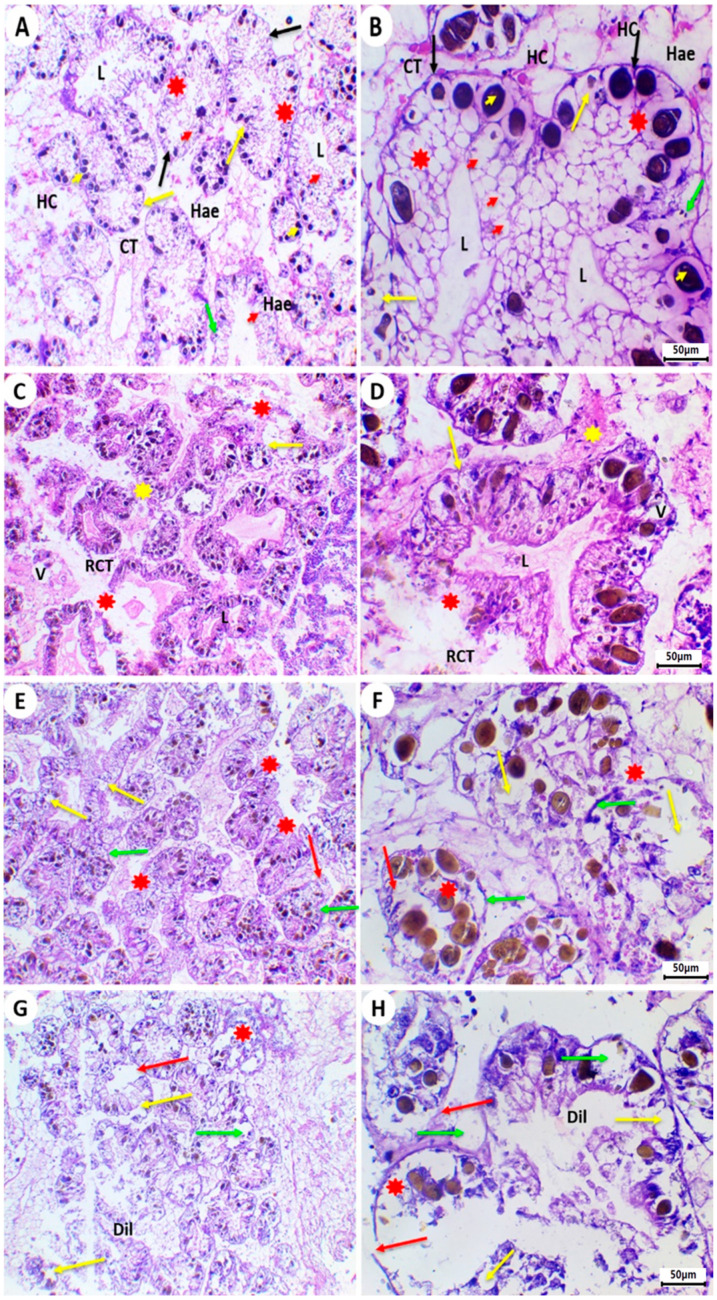
H & E-stained digestive glands of *L. carinatus*. (**A**,**B**) Digestive glands of the control *Lanistes carinatus* group showing normal structures with digestive tubules and secretory and digestive cells. Digestive tubules (red stars) with epithelial layers resting on a basement membrane (black arrows) and containing a narrow tubular lumen (L). Digestive tubules are connected to each other by normal connective tissues (CTs) between them and haemocoele (Hae) with hemocytes (HCs). Different types of cells are abundant, including digestive cells (red arrowheads), calcium cells (green arrows), and execratory cells (yellow arrows); in addition, dark stained corpuscles (yellow arrowheads) are clearly visible. (A = 10X, B = 40X). (**C**,**D**) Digestive glands of the *Lanistes carinatus* exposed to 1 mg/L PFOS showing degenerated digestive tubules (red stars), with congested lumens (L), ruptured connective tissues (RCTs), vacuolation (V), necrotic basement membranes (yellow arrows), and fused tubules (yellow stars). (H&E, C = 10X, D = 40X). (**E**,**F**) Digestive glands of the *Lanistes carinatus* exposed to 3 mg/L PFOS showing high degeneration (stars) and shrinkage of some tubules and cells that lost their regular shapes and became vacuolated (yellow arrows), ruptured villi (red arrows), and detached basement membranes of the digestive tubules (green arrows). (E = 10X, F = 40X). (**G**,**H**) Digestive glands of the *Lanistes carinatus* are exposed to 10 mg/L PFOS showing sever degeneration (stars) in digestive tubules including digestive cells, execratory cells, calcium cells and villi. In addition, rupture of the basement membranes of the digestive tubules (red arrows), and dilation (Dil) in tubular lumens (L) were observed. Also, different vacuolations (yellow arrows) and swelling cells (green arrows) were noted. (G = 10X, H = 40X).

Typically, the control group’s *L. carinatus* digestive glands are composed of tubules, each of which has a central lumen surrounded by a single layer of columnar epithelial cells that have undergone differentiation into digestive and secretory cells in addition to calcium cells. Digestive and secretory cells are located on the basement membrane and encircle a lumen in the middle of the tubule. The intertubular connective tissue binds the tubules together along with haemocoele fluid. There were many large-sized corpuscles in the basal third of the epithelial cells in the acidophilic cytoplasm of pyramidal cells (Figure 4A,B).

In the *L. carinatus* exposed to 1 mg/L PFOS, a range of deformities have been spotted. These damages include fused tubules, vacuolation, ruptured connective tissues, necrotic basement membranes, and degenerated digestive tubules with a clogged lumen (Figure 4C,D).

Following exposure to 3 mg/L PFOS, increased histopathological alterations have been observed in the digestive glands. These changes include ruptured villi, disconnected basement membranes of the digestive tubules, high degeneration and shrinking of some tubules, and cells that lost their normal morphologies and became vacuolated (Figure 4E,F).

Higher concentrations of PFOS (10 mg/L) caused the digestive glands of the *Lanistes carinatus* to exhibit severe degeneration in the digestive tubules, including the digestive cells, execratory cells, calcium cells, and villi, as well as the rupture of the digestive tubules’ basement membranes, dilatation of the tubular lumen, various vacuolations, and swelling cells (Figure 4G,H).

## 3. Discussion

During this study, we assessed the outcomes of PFOS exposure in the freshwater snail *L. carinatus* using a multi-marker approach. Snails were tested individually following a two-week exposure to 1.3 and 10 mg/L PFOS for biochemical markers, oxidative stress, and tissue damage. *L. carinatus* snails have become prominent sentinel models for ecotoxicological research during the last decades. Snails also play a significant role in tracking pollutants within aquatic environments [51]. Unfortunately, very little information has been published on the impacts of PFOS for these aquatic invertebrates in Egypt. When exposed to polluted water, contaminated food, or sediment, snails can absorb toxic substances through their skin. Their ability makes them useful as bioindicators of environmental pollution, as they can accumulate toxins in their tissues [62]. Accordingly, the present assessment focused on short-term exposure by examining the impacts of PFOS on antioxidant defenses such as (LPO, MDA, GST, CAT, GPx, GSH, SOD, and AchE). These indicators were selected for their ability to compensate for oxidative impacts of diverse environmental pollutants in previous investigations. However, oxidative stress can activate these defenses and thereby alter compensating mechanisms [63]. Several biological markers are widely employed in toxicity studies in freshwater snails [64].

Biomarkers are commonly employed in invertebrate toxicology to assess contaminant effects [65]. Specifically, measuring the impairment of cellular defenses offers valuable insights into how oxidative stress, lipid peroxidation (LPO), and apoptosis contribute to toxicity [66]. Malondialdehyde (MDA), a key product of LPO, serves as a widely recognized marker for oxidative damage; elevated MDA levels indicate the presence of reactive oxygen species (ROS) [67]. Antioxidants such as CAT and SOD counteract ROS to preserve cellular redox balance. Consequently, alterations in ROS levels, MDA concentrations, and inhabited antioxidant enzymes (e.g., CAT, SOD) are sensitive signals for inferring the harmful impacts of pollutant exposure [48,68].

PFASs are well-established inducers of oxidative stress [58]. Numerous studies confirm that PFAS exposure triggers oxidative stress in aquatic organisms, disrupting antioxidant enzyme activities (including CAT and SOD) and detoxification pathways [57]. The selection of 1 µg/L as the lowest test concentration was informed by documented environmental PFOS levels in surface waters (0.04–2709 ng/L), which vary based on factors like industrial proximity and wastewater discharge [69,70]. Higher concentrations were included to elucidate dose-dependent effects. Recent research, particularly on marine mussels, has demonstrated the impact of various PFASs on these oxidative stress biomarkers [71]. While snails naturally regulate reactive oxygen species (ROS) at low, stable concentrations under physiological conditions, chemical contaminants disrupt this balance, leading to the overproduction ROS, such as superoxide anions (O_2_^−^), hydroxyl radicals (OH), and H_2_O_2_. Inefficient removal of these ROSs overwhelms cellular defenses, resulting in macromolecular damage and lipid peroxidation [72]. We found that PFOS, in a dose-dependent manner, increases MDA content in *L. carinatus*: 10.0% at 1 mg/L, 32.0% at 3 mg/L, and 58.4% at 10 mg/L. This elevation suggests PFOS triggers oxidative stress in this species. Similarly, freshwater ray-finned fish *Pimephales promelas* exposed to PFOS showed substantial elevations in MDA content [60]. Increased MDA levels in snails exposed to contaminants strongly suggest that animals were harmed by excessive ROS [73,74].

PFOS can pose oxidative impacts and various histopathological problems, including changes in animal behavior, depending on the species and exposure levels [75,76]. LPO levels reflect the severity of intracellular damage and are commonly used to assess oxidative stress. Snail MDA content was utilized to evaluate LPO [77]. LPO remarkably increased progressively by exposure to PFOS showing a 16.3% increase at 1 mg/L, 39.6% at 3 mg/L, and 67.5% at 10 mg/L.

Two powerful antioxidant enzymes, SOD and CAT, form part of the antioxidant system, which resists the oxidative stress triggered by exogenous agents. SOD is considered the primary defense mechanism against reactive oxygen species. Under the catalysis of SOD, the O_2_ is first disproportionated into H_2_O_2_, and subsequently converted to H_2_O and O_2_ by CAT [78,79]. Our results indicate that following a 14-day period of exposure, the activity of SOD in *L. carinatus* treated with PFOS declined substantially compared with the control samples. Decreased enzyme activity may suggest that the antioxidant capacity has been exceeded by the concentration of hydroperoxide products from lipid peroxidation, detectable in the LPO levels of these tissues. After seven days of receiving a mixture of perfluorinated compounds [80], SOD activity in the snail *Perna viridis* exhibited higher records at lower concentrations (0–100 µg/L), while dropping at the highest levels (100–10,000 µg/L). Also, in *Unio ravoisieri*, the mean values of SOD were higher in groups treated with higher PFOS levels (2–6 mg/L) but declined in those exposed to 10 mg/L [57] for one week. SOD activity in the amphipod (*Gammarus insensitives*) increased and then decreased during four days at PFOS doses of 1 mg/L, 1.6 mg/L, and 3.1 mg/L [81]. Previous experiments have shown that the crab *Eriocheir sinensis* subjected to 10 mg/L PFOS for 21 days had decreased SOD activity [82] and over the course of seven days in *D. magna* exposed to PFOS or PFNA doses ranging from 0.008 to 5 mg/L [83].

In *L. carinatus*, CAT activity rose dramatically after 14 days of PFOS exposure compared with controls. Similar increases were noted in the freshwater mussel *Unio ravoisieri* subjected to 2–10 mg/L PFOS [57], and in *Mytilus galloprovincialis* treated with 1, 10, and 100 µg/L PFOA [84]. Furthermore, *P. viridis* displayed elevated CAT levels after 7 days of exposure to perfluorinated compounds (≤100 µg/L) [80]. *O. niloticus* similarly showed a significant increase in CAT activity following a 24 h treatment with 1, 5, 15, 30 mg/L PFOS or PFOA [59]. However, studies found decreased CAT activity in medaka fish (*Oryzias latipes*) liver following a 7-day exposure to 10, 50, or 100 mg/L PFOA [85], and in *D. magna* water fleas exposed to 0.04 mg/L PFOS or PFNA [83]. The findings show that species-specific variability and sensitivity in the perfluorinated compound-induced response in CAT activity are likely due to variations in their antioxidant potency. For hydroperoxide detoxification, GPx is the most significant peroxidase [86], where it converts H_2_O_2_ and organic hydroperoxides into water and alcohol.

In the present study, GPx levels increased in *L. carinatus* snails by 10.0% at 1 mg/L, 31.9% at 3 mg/L, and 58.4% at 10 mg/L, after 14 days of PFOS exposure compared with controls. Similarly, exposure to PFOA induced GPx activity in bivalves. *Mytilus edulis* exhibited a significant GPx increase, primarily at 200 μg/L, after 7 days [87]. In the clam *Ruditapes philippinarum*, a drastic rise in both GPx and peroxidase activity occurred following a 21-day exposure to PFOA (0.2, 2, 20 μg/L) [88]. Consistent with our findings, zebrafish (*Danio rerio*) exhibited elevated GPx levels after 96 h of exposure to 0.4–1.6 mg/L PFAS [89]. Conversely, studies reported suppressed GPx activity in the planarian *Dugesia japonica* following a 14-day exposure to 0.5–20 mg/L PFOA [90], and in the clam *Scrobicularia plana* exposed to 1 mg/L PFOS [91]. Interactions between PFAS and co-pollutants like microplastics also impact mollusk antioxidant responses. For instance, O’Donovan, Mestre [91] reported that exposure to microplastic-adsorbed PFOS altered the antioxidant system in *Scrobicularia plana*, increasing GPx activity in gills after 7 days [91]. This elevation is a common component of the oxidative stress response triggered by PFOS. The most well-known function of GST is its ability to catalyze the conversion of reduced glutathione (GSH) to xenobiotic compounds for detoxification [92].

By altering the availability of glutathione, this improves antioxidant defenses by making dangerous substances less reactive and easier to remove [93]. In *L. carinatus*, PFOS exposure significantly increased GST activity after 14 days. Similarly, elevated GST activity was observed in various species exposed to PFAS, including *M. galloprovincialis* mussels (digestive gland, PFOS/PFOA) [84], *Dugesia japonica* planarians (PFOA) [90], and *Melanotaenia fluviatilis* rainbowfish gills and liver (PFOA) [94]. Notably, transgenerational exposure in *D. magna* offspring also induced significantly higher GST levels from the second to fourth generations, indicating increased oxidative stress and adverse effects [95,96]. Conversely, GST inhibition occurred in *O. niloticus* hepatocytes (PFOS/PFOA) [59]. This species-, tissue-, and generation-specific variability underscores the complex role of GST in aquatic organism detoxification and antioxidant defense. GST induction, particularly at high contaminant concentrations as seen in mussels [97], suggests an adaptive detoxification response aimed at mitigating accumulation through enhanced biotransformation and excretion.

It is evident from the current study that MDA levels and GSH content are inversely related. Since GSH functions as a reducing agent and free radical scavenger, it is one of the most crucial components defending against oxidative attacks by ROS, including lipid peroxidation [98]. Unlike the variable responses of GST, CAT, and GPx, GSH depletion showed a strong positive linear correlation with PFOS exposure concentration. This dose-dependent decline establishes GSH as an excellent biomarker for quantifying oxidative stress in PFOS-exposed snails.

In addition to affecting critical antioxidant systems, such as glutathione and catalase, PFAS exposure also disrupts essential neurochemical processes. An essential enzyme for biological nerve conduction between cholinergic synapses is AchE [99]. Exposure to PFAS raises AchE levels and overexcites nerve cells, which impacts AchE activity. Furthermore, it alters the expression of genes involved in the development of nerve cells, which leads to fewer nerve cells and less branching, ultimately impairing nerve cell functioning [96]. In similar study on the planarian *Dugesia japonica*, AchE activity was altered following exposure to PFOS [90]. This result may be explained by the significantly accumulating acetylcholine in the fish tissues described by Ahammad Sahib, Sailatha [100], which is caused by AchE inhibition during pesticide stress, which decreases with exposure duration. Consistently, the current data matches those found in the published literature. Liang, Zhou [28] resulted that long-term exposure to PFOS *D. magna* inhibited the amount of cholinesterase (ChE). AchE activity significantly increased in *D. magna* exposed to PFOS; however, it was found that the organism downregulated AchE during non-exposure and elevated it during exposure [95].

Histopathology serves as a promising tool in ecotoxicology for evaluating tissue-level toxicity. The digestive gland of mollusks is primarily responsible for xenobiotic detoxification, metabolic regulation, immune defense, and homeostasis [101], making it a key focus in toxicology studies [102,103]. Our analysis showed substantial damage in snail digestive glands caused by PFOS. The control specimens displayed typical histological architecture without any pathological changes (Figure 4A,B). In contrast, PFOS exposure caused concentration-dependent damage including lesions to the digestive tubule components, such as digestive, excretory, and calcium cells, as well as villi, along with basement membrane ruptures, tubular lumen dilations, and cellular vacuolation and swelling (Figure 4C–H). The degeneration of these tubules is consistent with known reactions to foreign substances and may compromise digestive function [104,105]. Previous research has shown degeneration of digestive tubules after exposure to toxic substances [106]. These pathologies also correlate with molluscan inflammatory responses, which are validated biomarkers of toxicant exposure [107,108].

## 4. Materials and Methods

### 4.1. Chemicals

Perfluorooctanesulfonic acid potassium salt (PFOS; CAS No. 2795-39-3; molecular weight 538.22 g/mol; purity ≥ 98%; Product No. 77282) was sourced from Sigma-Aldrich (St. Louis, MO, USA). To make a stock solution, PFOS was dissolved in dimethyl sulfoxide (DMSO; final concentration in exposure water 0.01%) and stored at 4 °C in the dark to prevent degradation. Working solutions of the desired concentrations were freshly prepared daily by diluting the stock with dechlorinated tap water. All glassware and containers used in the preparation were rinsed thoroughly with methanol and ultrapure water to minimize contamination.

### 4.2. Experimental Animals

*L. carinatus* snails were collected from the Nile River at Assiut, Egypt, then transported to the laboratory in Nile water. To adapt, snails were kept in tanks with filtered, dechlorinated tap water for two weeks after collecting. In a laboratory environment, the snails were housed in water with a pH of 7.2 and a 12 h light–dark period. Following acclimation to the laboratory conditions, *L. carinatus* snails of similar size (7 ± 0.5 gm) were divided into glass tanks of four groups (three replicates for each/15 individual for each aquarium): one control group and three (1, 3 and 10 mg/L) PFOS concentration groups. Snails were fed lettuce during the 14-day experiment, and the aquarium’s water was replaced with a corresponding PFOS-continuing volume.

The selected concentrations of PFOS (1, 3, and 10 mg/L) were based on documented levels in contaminated aquatic ecosystems and previous ecotoxicological studies that successfully demonstrated dose-dependent oxidative, biochemical, and histopathological alterations in aquatic invertebrates and fish models [61,109,110]. Although the highest concentration (10 mg/L) exceeds typical environmental levels, such concentrations are commonly employed experimentally to simulate acute exposure scenarios, define worst-case thresholds, and uncover mechanistic pathways of toxicity. In highly contaminated freshwater systems near industrial and wastewater discharges, PFOS concentrations ranging from the µg/L scale to mg/L levels have been reported [61,109,110,111], with potential for bioaccumulation and trophic transfer leading to elevated internal burdens. Thus, the lowest concentration tested (1 mg/L) reflects a realistic sublethal exposure level in polluted waters, while higher doses enable the assessment of concentration-dependent responses in *Lanistes carinatus*. This concentration range therefore provides a balanced evaluation of both environmentally relevant and mechanistically informative exposure levels.

After 14 days, six snails were selected randomly from each treatment including control. For histopathological investigations, snails were separated from shells and dissected. Hemolymph was extracted for biochemical analysis using a fine needle and sterile 10 mL. Following collection, samples were centrifuged at 4000 rpm for 10 min to separate the cell-free supernatant. All hemolymph cell-free supernatant was stored in sterile Eppendorf tubes at −20 °C for later use. Six biological replicates per group were analyzed.

### 4.3. Antioxidant Enzymes

CAT, GPX, SOD, and GST were assessed using standard methods. CAT activity was determined following the protocol by Aebi [112] in accordance with the manufacturer’s instructions [112]. GPX level was recorded using a commercial assay kit according to the provided protocol. SOD was measured using the clinical assay adopted from Sun, Oberley [113]. GST activity was quantified using a microplate method containing 190 μL of sodium phosphate buffer, 200 mM of reduced glutathione, and 1-chloro-2,4-dinitrobenzene, as outlined by Valenzuela-Jiménez, Durruty-Lagunes [114].

### 4.4. Oxidative Stress

Lipid peroxidation (LPO) levels were determined using the PeroxiDetect© kit (Sigma-Aldrich, St. Louis, MO, USA) following the method of Fox, Blow [115]. Malondialdehyde (MDA) content, a marker of LPO, was measured at a wavelength of 535 nm using a UV–VIS spectrophotometer based on the thiobarbituric acid reaction described by Ohkawa, Ohishi [116] and modified by Hamed, Soliman [117]

### 4.5. Neurotoxicity Biomarker

Ellman method was followed to determine the activity of AchE, using the Fish AchE ELISA Kit (cat. no. MBS035436, BioSource, San Diego, CA, USA) [118,119].

### 4.6. Histopathology

From each group, six samples of *L. carinatus* digestive glands were taken for histological analysis, and they were fixed in Davidson’s fixative for an entire day [120], and then transferred to 70% ethanol. Tissues were then slowly treated with ethyl alcohol before being imbedded in paraffin wax. H&E staining was used after paraffin blocks were sliced into 5 μm sections [121]. The slides were examined under a microscope after staining.

### 4.7. Statistical Analysis

All output data are presented as mean ± SD, after statistical processing by the GraphPad Prism software version 9. Following ANOVA, Fisher’s least significant difference (LSD) post hoc test was applied to test and compare the differences between groups at a *p*-value of 0.05. Significant differences between control and treated groups are marked by asterisks, where * *p* < 0.05, ** *p* < 0.01, *** *p* < 0.001, and **** *p* < 0.0001.

## 5. Conclusions

As far as we know, this work presents a unique comprehensive assessment of PFOS-related ecological risks in *L. carinatus*. Our integrated analysis of neurotoxicity, lipid peroxidation, and antioxidant responses revealed that PFOS exposure in *L. carinatus* induces oxidative stress, characterized by higher MDA levels, depleted GSH content, dysregulated antioxidant enzymes (CAT, GPx, GST), and hindered AchE activity. Histopathological alterations in the digestive gland were also observed. These findings provide critical insights into environmental policy, supporting enhanced PFOS regulation and monitoring in aquatic ecosystems especially in high-aquatic food-consumption regions. Aquatic PFOS levels must be strictly limited to lessen ecological and human health issues. Furthermore, preventive actions, remediation plans, and health protection programs are required to accomplish thorough pollution management. This study enhances our comprehension of the biochemical impacts of PFOS on aquatic species, thereby supporting a more comprehensive approach to tackling the difficulties presented by long-lasting environmental pollutants. Future studies should investigate underlying mechanisms across additional perfluorinated compounds and species to inform comprehensive environmental protection strategies.

## Data Availability

Data will be available upon request from the corresponding author.

## Abbreviations

PFOS	Perfluorooctane sulfonate
PFAS	Per- and polyfluoroalkyl substances
PFOA	Perfluorooctanoic acid
C-F	Carbon–fluorine (bond)
LC_50_	Lethal Concentration for 50% of test organisms
LPO	Lipid peroxidation
MDA	Malondialdehyde
GST	Glutathione S-transferase
GPx	Glutathione peroxidase
CAT	Catalase
GSH	Reduced glutathione
SOD	Superoxide dismutase
AchE (or AChE)	Acetylcholinesterase
ROS	Reactive oxygen species
POD	Peroxidase
GR	Glutathione reductase
ChE	Cholinesterase
DMSO	Dimethyl sulfoxide
H&E	Hematoxylin and eosin (stain)
SD	Standard deviation
ANOVA	Analysis of variance
LSD	Least significant difference (test)
*L. carinatus*	*Lanistes carinatus* (snail species, used in short form)
µg/L, mg/L, ng/L	Micrograms/milligrams/nanograms per liter
